# Diversity of Parasitic Diarrhea Associated with *Buxtonella Sulcata* in Cattle and Buffalo Calves with Control of Buxtonellosis

**DOI:** 10.3390/ani9050259

**Published:** 2019-05-21

**Authors:** Saeed El-Ashram, Shawky M. Aboelhadid, Asmaa A. Kamel, Lilian N. Mahrous, Khatib H. Abdelwahab

**Affiliations:** 1 School of Life Science and Engineering, Foshan University, Foshan 528231, Guangdong, China; 2 Faculty of Science, Kafrelsheikh University, Kafr el-Sheikh 33516, Egypt; 3 Department of Parasitology, Faculty of Veterinary Medicine, Beni Suef University, Beni-Suef 62511, Egypt; drasmaaalaa@yahoo.com (A.A.K.); Lilian_nagy@yahoo.com (L.N.M.); 4 Minya Vet. Administration, Minya 61111, Egypt; khateebabdelwahab704@gmail.com

**Keywords:** diarrhea, suckling and post-weaning calves, cattle, buffaloes, *Buxtonella sulcata* multiple infections, *B. sulcata* mono-infection

## Abstract

**Simple Summary:**

We investigated the relationship between parasitic infections as a cause of diarrhea in suckling and post-weaning cattle and buffalo calves and *Buxtonella sulcata* infection, which remains an important pathogenic factor of diarrhea in Egypt. *B. sulcata* mono-infection was found more frequently associated with other parasitic infections that cause diarrhea. Drug treatment exhibited a higher efficacy than treatment with garlizine, a natural product in the treatment of buxtonellosis.

**Abstract:**

The association between parasite isolates, including *Buxtonella sulcata*, in suckling and post-weaning calves and diarrhea was studied with the aim to control diarrhea caused by *B. sulcata*. A total of 1100 diarrheic fecal samples were collected from 609 suckling calves and 491 post-weaning calves with diarrhea. Salt floatation and modified Ziehl–Neelsen techniques were applied for the microscopic examination of the presence or absence of parasite eggs and oocysts/cysts. The microscopic findings revealed that 20.36% of the calves had parasitic diarrhea, with a prevalence rate of 19.54% in suckling calves and 21.38% in post-weaning calves. The most frequently detected parasites according to morphological characters were *Eimeria* species, *Buxtonella sulcata*, *Toxocara vitulorum*, *Cryptosporidium* species, and *Moneizia* species. In suckling calves, *Eimeria* species, *B. sulcata,* and *T. vitulorum* had the highest prevalence rates of infection, corresponding to about 37.14%, 32.86%, and 20.00%, respectively. However, in post-weaning calves, *B. sulcata* infection was more prevalent (30.15%) than infections with *Eimeria* species and *T. vitulorum*. The highest parasite score density was found in multiple infections with *B. sulcata, Eimeria* species, and *T*. *vitulorum;* however, the score density of *B*. *sulcata* when present alone in the fecal specimens was higher than in specimens co-infected with other parasites. The risk factors affecting the prevalence rate of parasitic diarrhea, such as sex, season, housing system, and feed stuff, are discussed. Concerning the treatment of diarrhea caused by *B. sulcata* in post-weaning cattle calves, 20 calves were divided into 4 equal groups. Group A was given sulphadimidine sodium (1.0 g/10 kg body weight) and metronidazole (500 mg/40 kg body weight); group B was treated with oxytetracycline hydrochloride (500 mg/45 Kg of body weight) and metronidazole (500 mg/40 kg body weight); group C was daily administered garlizine (allicin), 2 g/ L in drinking water; group D was the untreated control group. All medications were administered orally for four successive days. The results showed that the cyst count was significantly lower in the drug-treated groups, and the metronidazole + oxytetracycline hydrochloride and metronidazole + sulphadimidine combinations achieved 98.77% and 96.44% efficacy, respectively. Garlizine had 72.22% efficacy. Intriguingly, *B. sulcata* infection was associated with other parasitic infections, but *B. sulcata* mono-infection was the most common cause of diarrhea. Moreover, the combinations of oxytetracycline hydrochloride or sulphadimidine with metronidazole are recommended to control buxtonellosis in calves. Further studies are recommended to investigate the bacterial, viral, and fungal infections associated with *B. sulcata* infection.

## 1. Introduction

Parasitic diarrheal disease continues to be a major cause of morbidity and mortality in the developing world. Neonatal and young calves are highly susceptible to enteric infections by various pathogens, including bacteria, viruses, fungi, protozoa, and helminths [1]. The most commonly identified parasitic agents responsible for diarrhea are: *Cryptosporidium* spp., *Eimeria* spp., *Giardia* spp., *Toxocara vitulorum*, and *Buxtonella sulcata* [2]. However, bacterial, viral, fungal agents, and nutritional factors also play a role in causing diarrhea [3,4]. Diarrhea may occur as a consequence of stress, improper sanitation, or sudden feed changes [3,5,6]. *Cryptosporidium* is one of the most common enteropathogens present in calves during the first two weeks of age [7]. *Cryptosporidium* infection in calves is characterized clinically by non-specific diarrhea, dehydration, depression, anorexia, and abdominal pain. In most calves, diarrhea starts 3–5 days post-infection and lasts from 4 to 17 days [7,8].

Bovine coccidiosis is one of the most common parasitic diseases of cattle currently occurring as a subclinical disease, which causes great economic losses [9]. Coccidial infection affects all age groups but is most common and important in young animals [10,11]. *T. vitulorum* is a nematode living in the small intestine of cattle and water buffalo calves and may also cause diarrhea together with anemia, weight loss, and anorexia in calves aged 1–3 months [12]. The infection rate is the highest in 1- to 3-month-old calves and decreases as the animal gets older [13,14]. *B. sulcata* is a ciliate with two stages: trophozoite and cyst stages. The trophozoites colonize and invade the colonic wall of cattle and cause diarrhea in calves [2]. The prevalence rate of diarrhea was substantially higher in calves having *B. sulcata* cysts than in those without the cysts [15]. In several studies, *B. sulcata* infection in ruminants is associated with diarrheal symptoms [2,16,17]. However, the association between *B. sulcata* infection and other parasitic diseases contributing to diarrhea remains to be investigated. Therefore, in the present study, we tried to investigate the impact of single- and multiple-species parasitic infections on diarrhea in suckling and post-weaning calves. Treatment of buxtonellosis was carried out, and the overall efficacy of oxytetracycline and metronidazole in cattle was 66% and 33%, respectively [18]. In a previous study, metronidazole, oxytetracycline, and secnidazole were used for the treatment of *Balantidium coli* infection in cattle with efficacies of 37.5%, 62.5%, and 87.5%, respectively [19]. Similarly, oxytetracycline was selected as an antibacterial and antiprotozoal agent for persistent diarrhea in a heifer calf [20]. Garlic has antiparasitic (antihelminthic) [21], antiprotozoal [22], and acaricidal properties [23]. Garlic activity is based on allicin, which is the main constituent of garlic [24,25]. Thus, combinations of antimicrobials with antiprotozoal agents were tested to control buxtonellosis in calves.

## 2. Materials and Methods

### 2.1. Study Area

This study was conducted in different localities in El-Minia province (GPS coordinates: N 28°06′35.57″, E 30°45′1.08″), Egypt, from May 2016 to April 2017 to determine the prevalence rate of parasitic diarrheal agents among suckling and post-weaning calves (cattle and buffalo).

### 2.2. Diarrheic Fecal Sample Collection

A total of 1100 calves (cattle and buffalo) were brought to the veterinary clinics in El Minya province because they suffered from loss of appetite, significant loss weight, pale yellow mucous membranes, and presence of diarrhea. The calves included 609 suckling animals (353 cattle and 256 buffaloes) and 491 post-weaning calves (267 cattle and 224 buffaloes). The veterinarians asked the owners about housing system, feeding, watering, and animal density in their farm or animal house. Two samples were collected from each diarrheic case at the veterinarian clinic, and about 5–10 g was directly collected from the rectum of each animal using disposable plastic gloves. All fecal samples were collected in separate cups to prevent potential cross contamination between fecal samples. Fecal samples were placed in labeled clean plastic cups and preserved in an ice tank before transporting to the Parasitology Laboratory, Faculty of Veterinary medicine, Beni-Suef University. The date of collection, age, sex, and owner’s name were recorded. Fecal samples, which were collected during field surveys, were stored at 4 °C for a maximum of two days until further processing [26].

### 2.3. Examination Techniques

The physical appearance of the fecal samples was examined macroscopically, while intestinal parasites were examined microscopically [6]. A direct fecal smear for protozoal vegetative forms, cysts, and helminth eggs from each fecal sample was analyzed. The salt floatation technique was performed as described by Zajac and Conboy [27], El-Ashram et al. [28], and El-Ashram and Suo [29]. Briefly, 5 g of fecal sample was mixed with a little amount of saturated salt solution (NaCl) and homogenized with a pestle and mortar. The mixture was then sieved into a flat-bottom floatation tube, which was filled up to the top with the saturated salt solution. The tube was covered with a glass slide, which was removed after 20 min, and examined microscopically under high power for the presence of intestinal parasite eggs, cysts, and oocysts. A modified McMaster technique was used to determine fecal oocyst counts [27]. Furthermore, the modified Ziehl–Nelseen technique was applied for the detection of *Cryptosporidium* oocysts [30]. Parasite identification was based on the clinical signs, including physical appearance of feces and presence or absence of adult worms or tapeworm segments. This diagnosis was confirmed by microscopic examination of the fecal samples [6]. The prevalence rate for each parasite was calculated using the following formula:

Prevalence rate (%) = Number of infected individuals (n) × 100(1)

Total number of sampled individuals (N).

### 2.4. Trial for Control of Parasitic Diarrhea

Twenty post-weaning cattle calves suffered from greenish offensive-odor diarrhea with arched-back position, pale yellow mucous membranes, and significant weight loss with no fever. The experiment was conducted during the beginning of Berseem, Egyptian clover (*Trifolium alexandrinum*) season in Egypt (November). Fecal examination was carried out according to Tomczuk et al. [2] to record the presence of cysts or trophozoites of *B. sulcata*.

All examined cases had cysts of *B. sulcata* only (i.e., no other parasites were detected microscopically). The number of cysts per gram of feces (CPG) was calculated from the formula:

CPG = number of cysts in both parts of McMaster chamber/5 × 100.(2)

The twenty post-weaning cattle calves were divided into four groups (five animals in each group). The calves were subjected to treatment of parasitic diarrhea as follows: group A was treated orally by a combination of sulphadimidine sodium in a powdered form (Aveco, Egypt) (1.0 g/10 kg body weight) and metronidazole tablets (El Nile Company, Giza, city, Egypt) (500 mg/40 kg body weight); group B was administered a combination of oxytetracycline hydrochloride (HCL) in a powdered form (powder, Aveco, Egypt) (500 mg/45 Kg of body weight) and metronidazole tablets (500 mg/40 kg body weight); the third group C was received a daily dose of garlizine (allicin) (Pharma Swede Egypt) 2 g/L in drinking water. All medications were administered orally for four consecutive days. The last group D was left untreated and served as a control group. The animals in all groups were subjected to fecal examination and counting of cysts daily for two weeks after treatment. The efficacy of each medication regimen was calculated by the following equation:

Cyst number in the untreated control group − cyst number in the treated group × 100(3)

Cyst number in the untreated control group.

### 2.5. Statistical Analysis

All data were coded, entered, and analyzed using the statistical package SPSS version 22 (IBM Corp. Released 2013. IBM SPSS Statistics for Windows, Version 22.0. Armonk, NY: IBM Corp. USA). The data were summarized using a descriptive frequency and percentage for quantitative values. The relations between data grouped were tested by the Chi-square test for quantitative variables, and *p*-values were calculated. The treatments data were analyzed using one-way ANOVA followed by Duncan’s multiple range test to compare the treatments means, and the results were expressed as means and standard error of the means (SEM). For all the above-mentioned statistical tests, *p*-values less than or equal to 0.05 (*p* ≤ 0.05) were considered significant.

The prevalence rate for each parasite was calculated using the following formula:

Prevalence rate (%) = Number of infected individuals (n) × 100(4)

Total number of sampled individuals (N).

## 3. Results

### 3.1. Overall Prevalence Rate of Intestinal Parasitic Diarrhea Among Cattle and Buffalo Calves

Fecal examination of 1100 diarrheic cattle and buffalo calves revealed that 224 (20.36%) calves (119 suckling and 105 post-weaning calves) were infected with different intestinal parasites. The prevalence rate of parasitic infection among the diarrheic suckling calves was 19.54%, while the prevalence rate was 19.83% in cattle calves and 19.14% in suckling buffalo calves. Moreover, the infection rate among the post-weaning calves was 21.38%, (infected 105/491 diarrheic calves). The infection rate in cattle calves was 23.22% and 19.19% in post-weaning buffalo calves. There were no significant differences between animals with respect to their ages and species (Table 1).

### 3.2. Parasites among Diarrheic Cattle and Buffalo Calves

**Figure 1:**
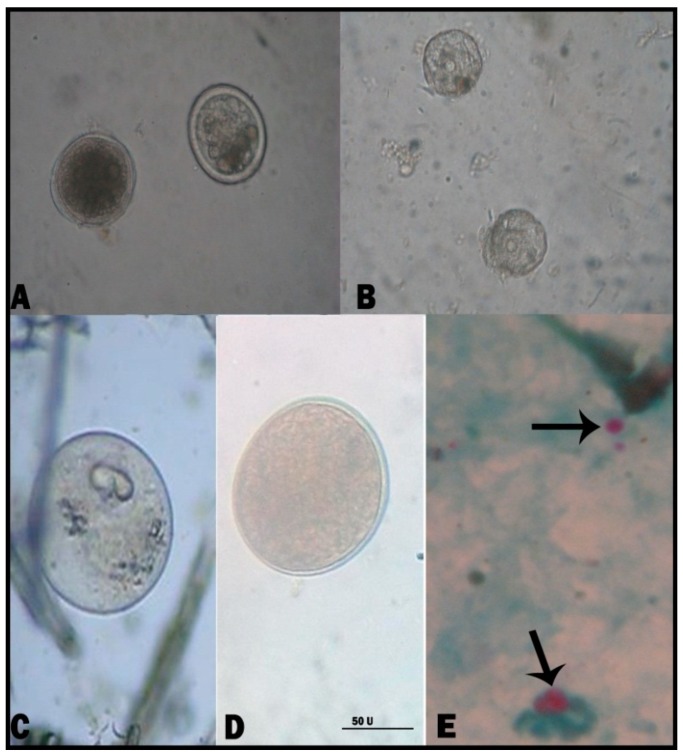
Diarrhea-causing intestinal parasites in cattle and buffalo calves (Plate 1). (**A**) *T.* *vitulorum* egg; (**B**) *Moneizia* species egg; (**C**) *B. sulcata* (trophozoite); (**D**) *B.* *sulcata* (cyst form); and (**E**) *Cryptosporidium* species (oocyst). (Scale bar = 50 μm).

**Figure 2:**
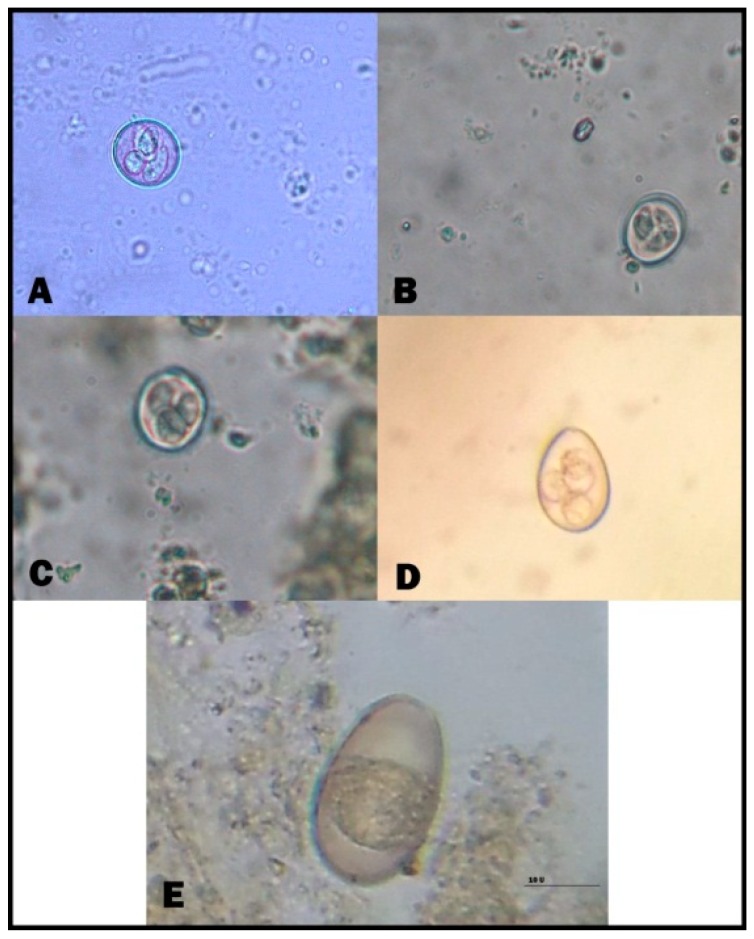
Diarrhea-causing intestinal parasites in cattle and buffalo calves. (**A**) *Eimeria zurnii* (sporulated oocyst); (**B**) *Eimeria ellipsoidalis* (sporulated oocyst); (**C**) *Eimeria alabamensis* (sporulated oocyst); (**D**) *Eimeria bovis* (sporulated oocyst); (**E**) *Eimeria bukidnonensis* (unsporulated oocyst). (Scale bar = 20 μm).

*B. sulcata*, *T. vitulorum*, *Eimeria* species, *Cryptosporidium* species, and *Moneizia* species were reported in the examined calves. In suckling calves, *Eimeria* species and *B. sulcata* recorded the highest infection rates, i.e., 37.14% and 40.82% for *Eimeria* species in cattle and buffalo calves, respectively, and 32.86% and 36.73% for *B. sulcata* in cattle and buffalo calves, respectively. The prevalence of *Cryptosporidium* spp. was the lowest among parasites that cause diarrhea in suckling calves. Moreover, *T. vitulorum* had infection rates of 20.00% and 12.24% in cattle and buffalo calves, respectively. There was no significant difference between different parasites in suckling calves. In post-weaning calves, the same parasites were still the cause of diarrhea, and *B. sulcata* was the most common cause of diarrhea, especially in cattle calves, at a rate of 36.73%. In addition, the helminths caused diarrhea, with an increased appearance of *Moneizia* spp. besides *T. vitulorum*. There was a significant difference regarding diarrhea-causing parasites in post-weaning calves (Table 2). Strikingly, *Eimeria* species infection was detected at a rate of 28.57%. The microscopical identification of eimerian oocysts revealed the presence of five eimerian species (namely, *Eimeria bovis*, *Eimeria zurnii*, *Eimeria ellipsoidalis*, *Eimeria alabamensis*, and *Eimeria* *bukidnonensis)* (Figure 1 and Figure 2, Supplementary Table S2). *E. bovis* and *E. zurnii* were the most prevalent species in suckling and post-weaning calves (cattle and buffalo).

### 3.3. B. Sulcata Infection Occurs in Association with Other Parasitic Infections

The prevalence rate of single *B. sulcata* infection was 15.6% (Table 3, Supplementary Table S2 and Figures S1 and S2). Furthermore, *Eimeria* species (21 cases) and *T. vitulorum* (12 cases) were the most common infections associated with *B. sulcata* (Table 4).

### 3.4. Risk Factors that Affect the Prevalence Rate of Parasitic Diarrhea

Regarding animal sex, males in suckling and post-weaning stages had higher prevalence rates than females for both cattle and buffalo calves in a non-statistically significant manner Supplementary Table S3. Additionally, the housing system had no significant effect on the prevalence rate of diarrhea in our study Supplementary Table S4. Our results showed that the feeding system had a significant effect on the prevalence rate of parasitic diarrhea in calves (*p* < 0.05). If the suckling calves fed on breast milk, there was a significantly high (*p* < 0.05) risks of infection (37.14% cattle and 40.81% buffalo). Post-weaning calves fed on green fodder had significantly higher (*p* < 0.05) infection rate than those fed on dry mix (Table 5). The seasonal effect on infection showed no significant difference between different seasons Supplementary Table S5.

### 3.5. Treatment Trial for Buxtonellosis

After one week of treatment, the results showed the absence of severe diarrhea. Additionally, two weeks post-treatment, a marked improvement of the condition in the treated calves was clearly observed. Microscopic examination revealed a sharp decline of cyst count (*p* < 0.05) two weeks post-treatment (Table 6). The efficacy of the sulphadimidine + metronidazole and oxtetracycline + metronidazole combinations (groups A and B) reached 96.44% and 98.77%, respectively. The garlizine-treated group exhibited an obvious improvement in fecal consistency (i.e., separate hard lumps); however, the cyst number was significantly higher than in the drug-treated groups (*p* < 0.05), though still lower than in the untreated control group (Table 6).

## 4. Discussion

Parasitic diarrhea of calves is a commonly reported disease in young animals. In the current study, fecal examination of 1100 diarrheic calves (cattle and buffaloes) revealed that 224 (20.36%) calves were positive for different intestinal parasites. The prevalence rate of parasitic diarrhea among suckling calves (1–60 d) was 19.83% and 19.14% in cattle and buffaloes, respectively. Moreover, the rate of infection among the post-weaning calves (2–6 m) was 23.22% and 19.19% in cattle and buffaloes, respectively. Similar findings were obtained by Rana et al. [31] who reported a prevalence rate of infection 16.10% (24/142) in neonatal buffalo calves in Hisar district, Haryana. Our prevalence rates were lower than those previously reported in the literature [32,33,34]. Herein, the most common parasites found in calves were *Eimeria* species, *B. sulcata*, *Cryptosporidium* species, and *T. vitulorum*. These results are in agreement with those obtained by El-sherif and Aboelhadid [35], Reberio et al. [36], GÖZ et al. [32], and Ramadan et al. [34].

In the current investigation, *Eimeria* species infection was the most common among suckling calves (37.14% and 40.82% prevalence rates in cattle and buffaloe calves, respectively). These findings are in line with those reported by El-sherif and Aboelhadid [35] in Egypt (10.50%) and Heidari et al. [37] in Iran (8.25%). In this respect, Reberio et al. [36] and GÖZ et al. [32] reported that the most common parasite in neonatal suckling calves was *Eimeria* species. Furthermore, different prevalence rates of *Eimeria* species were reported in different regions of the same country (Turkey), for example by Yüksekova-Hakkari (89.13%) [38] and Yüzüncü Yıl (69.8%) [39]. These variations could be attributed to seasonal, climatic, and geographical differences and management and husbandry practices of the studied animals in different regions.

The prevalence rate of *B. sulcata* ranks second to the most common parasite, *Eimeria* species, that causes diarrhea among the examined calves of both ages (32.86% and 30.15%). Some researchers revealed that *B. sulcata* can cause diarrhea in cattle and buffalo calves as a result of changes in the parasite microenvironment in the gastrointestinal tract (GIT), especially pH changes [2,15]. These results are in line with those of previous studies (Mamatho and Souza [40] and GÖZ et al. [32]). However, the rates we determined were lower than those reported by Sultan et al. [41], Al-Saffar et al. [42], and Tomczuk et al. [2] who recorded infection rates of 48.2% in cattle in Egypt and 24.1% in buffalo calves in Mosul province Iraq. These discrepancies in the percentage of infection may be due to the age of the animals, their health status, the number of cysts, and the intensity of infection. In addition, when the diet was changed from freshly cut Berseem (Egyptian clover) to conserved Berseem as silage with an increase in supplementary concentrate feeding, the carbohydrate intake by the animals increased, which might have affected *B. sulcata* population dynamics [42]. *Cryptosporidium* species were detected in 20.00% and 12.24% of 224 diarrheal cattle and buffalo calves, respectively. This values are lower than those reported by GÖZ et al. [32], Singh et al. [22] and Sharma and Busang [33], who detected *Cryptosporidium* spp. in young calves (0–3 months) at prevalence rates of 18.6%, 65.7%, and 30.9 ± 5.6%, respectively. This variation could be attributed to geographical differences, feeding systems, stress, improper sanitation, and technique of examination. *T. vitulorum* was a cause of diarrhea in young calves with prevalence rates ranging from 12.24% to 23.80% in cattle and buffalo calves, respectively, for both ages. These findings are in accordance with data published by Riberio, et al. [36] and Ramadan et al. [34], who stated that *T. vitulorum* was the most helminthic pathogen in diarrheic buffalo calves (17.2%), while its prevalence rate was 12% in cattle calves in Munofyia province, Egypt. This could be partially due to the existence of several routes of transmission, such as trans-placental and trans-mammary transmission or ingestion of larvated eggs. In addition, the data of the present study revealed that *Moneizia* species eggs were recorded in post-weaning calves combined with other intestinal parasites, including *T. vitulorum*, *Eimeria* species, and *B. sulcata*, while single-infection diarrheic calves had a mono-infection with *Moneizia* species.

At this stage of post-weaning age, the calves begin to eat roughage and green fodders, which may be contaminated with the orbatide mite (intermediate host of anoplocephalids), considered the source of *Moniezia* infection [6]. The study of the association between *B. sulcata* and other parasitic infections in this study revealed that the most common pattern of parasitic co-infection was a double infection with *B. sulcata* and *Eimeria* species and of *B. sulcata* with *T. vitulorum*. Meanwhile, single infections with *B. sulcata* (35 case) were more frequent than *B. sulcata* coinfections with other parasites, indicating that *B. sulcata* might be the cause of diarrhea. In fact, the number of protozoan pathogens in the feces was high (≤500 cyst per gram), which reflected an increase of *B. sulcata* invasion. This could contribute to the acceleration of alimentary content passage in the intestine, resulting in diarrhea [2,15]. Moreover, a sudden change in the feeding system affects the digestive tract pH, and an incorrect diet causes diarrhea in calves owing to the multiplication of *B. sulcata* and the enhancement of the parasite virulence [5]. Therefore, *B. sulcata* is a frequent cause of diarrhea in calves. However, the association of bacterial, viral, and fungal infections with *B. sulcata* remains to be investigated [43].

The risk factors in this study revealed that the age is one of the major risk factors in the spread of parasitic infections. Furthermore, morbidity and risk of infection are greater in younger animals than in aging animals [9]. Here, the infection rate was nearly 20%, which may be attributed to the fact that calves are more susceptible to parasitic diarrhea than adults because of their immature immune system [11,44,45,46]. In addition, calf gender had no significant effect on the infection. However, the findings of the current study do not support previous researches [36,42,47] that recorded higher prevalence rates of intestinal parasites in males than in females. Similarly, Priti et al. [44], Rehman et al. [12], and Jahanzaib et al. [48] recorded a higher prevalence of infections in female cattle and buffalos than in males.

The housing system had no significant effect on the infection rate by diarrheal pathogens. These results differ from other published studies by Ernst et al. [49], Rehman et al. [12], and Jahanzaib et al. [48], which reported that parasitic infections were more common in confined herds than in animals kept on pastures. The current investigation revealed that the feeding system had a great effect on the infection rate by diarrheal parasites (*p* < 0.05). For example, dry-mix feed materials were associated with the lowest infection rate among the examined calves. This result may be explained, for suckling calves, by the possible external contamination of udders and the contamination of milk containers and, for newly born calves, by the presence of a less-developed immune system. Similar findings were obtained by Abebe et al. [10] and Singh et al. [50], in contrast to earlier findings by Rodríguez-Vivas et al. [51], Waruiru et al. [52], Wahid and Soad [53], Rahmatullah and Kamboh [54], and Ramadan et al. [34]. No significant data were obtained in the present study concerning a seasonal effect on the infection rate.

Buxtonellosis in ruminants has not been deservedly studied, and most studies have concentrated on its prevalence. Previous studies have revealed a strong relationship between the intensity of infection (i.e., the number of cysts per gram of feces) and diarrhea in cattle [2,55]. Besides, in the current investigation, among the 224 parasitic diarrheic calves, 35 calves had a mono-infection with *B. sulcata* and did not respond to antibiotic treatment.

Interestingly, *B. sulcata* is not the primary cause of diarrhea, but its infection could be complicated by secondary bacterial infections, which increase the intensity of the clinical symptoms [2]. This study, therefore, set out to assess the effect of combinations of sulphadimidine with metronidazole or oxytetracycline HCL with metronidazole on buxtonellosis. Remarkably, data showed a complete absence of diarrhea and a significant reduction in the mean fecal cyst count (*p* < 0.05) after both chemical treatments.

In addition, animals in group C were administered garlizine as a natural treatment and experienced an improvement in fecal consistency. Furthermore, the cyst count was significantly lower than in the untreated control, though still higher (*p* < 0.05) than in the drug-treated groups. This finding is supported by Sivajothi and Sudhakara [56] who reported that a combination of oxytetracycline hydrochloride, metronidazole, and furazolidone showed a complete elimination of *B. coli* cysts or trophozoites by the 3rd day of therapy in buffalo calves. Meanwhile, a previous study by Hasheminasab et al. [19] recorded that oxytetracycline alone or metronidazole alone showed efficacies of 66% and 33%, respectively, against *B. sulcata*. Therefore, the combination of oxytetracylcines, metronidazole, and sulphadimidine are useful therapeutic agents against *B. sulcata* infection. Out of 1100 examined calves with diarrhea, 876 calves (79.63%) had no parasites. Furthermore, the combination of oxytetracylcine or sulphadimidine with metronidazole had improved efficacy against *B. sulcata* diarrhea. The efficacy of garlizine was lower than that of the above-mentioned drug treatments. Such treatment is safe and has no side effects [23,57].

## 5. Conclusions

Parasitic diarrhea in cattle and buffalo calves (suckling and post-weaning) in this study was due to *Eimeria* species, *B. sulcata*, *Cryptosporidium* species, *T. vitulorum*, and *Moniezia* species. The association between *B. sulcata* and other parasitic infections in this study revealed that the most common pattern of parasitic co-infection was a double infection with *B. sulcata* and *Eimeria* species followed by *B. sulcata* with *T. vitulorum*. Meanwhile, a single infection with *B. sulcata* (35 case) was more frequent than *B. sulcata* coinfections with other parasites, indicating that *B. sulcata* might be the cause of diarrhea. Therefore, *B. sulcata*-associated diarrhea was treated with combinations of oxytetracylcine or sulphadimidine with metronidazole, which showed improved efficacy against *B. sulcata* diarrhea compared to garlizine.

## Figures and Tables

**Table 1:** Age-wise prevalence rate of parasitic diarrhea in cattle and buffalo calves.

Species	Cattle Calves		Buffalo Calves		Total Parasitic Diarrhea	SEM	*p*-Value *
	Diarrheic Animals	Parasitic Diarrhea	Diarrheic Animals	Parasitic Diarrhea			
Suckling calves	353	70 (19.83%)	256	49 (19.14%)	119 (19.54%)	2.64	0.524
Post-weaning calves (below 6 months of age)	267	63 (23.22%)	224	42 (19.19%)	105 (21.38%)		
Total	620	133 (21.45%)	480	91 (18.95%)	224 (20.36%)		

Data are presented as number of positive animals, with prevalence rate in parentheses; * *p*-value > 0.05 is non-significant (NS).

**Table 2:** Prevalence rate of intestinal parasites causing diarrhea in cattle and buffalo calves.

Age Groups	Species	Prevalence Rate of Detected Parasites					SEM	*p*-Value
		*Eimeria* spp.	*Cryptosporidium* spp.	*Buxtonella Sulcata*	*Toxocara Vitulorum*	*Moneizia* spp.		
Suckling calves (1d–60d)	Cattle 70	26 (37.14%)	7 (10.00%)	23 (32.86%)	14 (20.00%)	0.00	3.01	0.487
	Buffalo 49	20 (40.82%)	5 (10.20%)	18 (36.73%)	6 (12.24%)	0		
Post-weaning calves (below 6 months of age)	Cattle 63	18 (28.57%)	3 (4.76%)	19 (30.15%)	15 (23.80%)	8 (12.69%)	0.47	0.001 *
	Buffalo 42	14 (33.33%)	3 (7.14%)	12 (28.57%)	8(19.04%)	5 (11.90%)		

Data are presented as number of positive animals, with prevalence rate in parentheses; * *p*-value > 0.05 is non-significant (NS), * *p*-value ≤ 0.05 is significant.

**Table 3:** Prevalence rate of mono-infections in diarrheic calves.

Age Groups	Species	*Eimeria* Species	*Cryptosporidium* Species	*B. Sulcata*	*T. Vitulorum*	*Moneizia* Species	SEM	*p*-Value *
Suckling calves (1–60 d)	Cattle 70	12 (17.14%)	3 (4.30%)	13 (18.60%)	6 (8.60%)	0	0.45	0.158
	Buffalo 49	13 (26.53%)	3 (6.12%)	13 (26.53%)	2 (4.08%)	0.00		
Post-weaning calves (below 6 months of age)	Cattle 63	7 (11.11%)	1 (1.58%)	5 (7.93%)	6 (9.52%)	0.00	0.28	0.296
	Buffalo 42	5 (11.90%)	3 (7.14%)	4 (9.52%)	3 (7.14%)	1 (2.38%)		
Total	224	37 (16.51%)	9 (4.02)	35 (15.63%)	17 (7.59%)	1 (0.45%)		

Data are presented as number of positive animals, with prevalence rate in parentheses; * p-value > 0.05 is non-significant (NS).

**Table 4:** Prevalence rate of *B. sulcata* co-infection with other parasites in diarrheic calves.

Age Groups	Species	*B. Sulcata + Eimeria* Species	*B. Sulcata* + *Cryptosporidium* Species	*B. Sulcata* + *Moneizia* Species	*B. Sulcata + T. Vitulorum*	SEM	*p*-Value *
Suckling calves (1–60 d)	Cattle 70	5 (7.14%)	1 (1.43%)	0.00	4 (5.71%)	0.4	0.441
	Buffalo 49	2 (4.10%)	1 (2.04%)	0.00	2 (2.08%)		
Post-weaning calves (below 6 months of age)	Cattle 63	8 (12.69%)	1 (1.58%)	1 (1.58%)	4 (6.34%)	0.52	0.286
	Buffalo 42	6 (14.28%)	0.00	0.00	2 (4.76%)		
Total	224	21 (9.35%)	3 (1.34%)	1 (0.45%)	12 (5.36%)		

Data are presented as number of positive animals, with prevalence rate in parentheses; * *p*-value > 0.05 is non-significant (NS).

**Table 5:** Prevalence rate of parasitic diarrhea in relation to feeding system.

Internal Parasites	Feeding System	Cattle Calves				Buffalo Calves				SEM	*p*-Value *
		Suckling Calves 70		Post-Weaning Calves 63		Suckling Calves 49		Post-Weaning Calves 42			
		No.	%	No.	%	No.	%	No.	%		
*Eimeria* species	Natural milk	26	37.14	-	-	20	40.81	-	-	0.88	0.000
	Green fodder	-	-	12	19.04	-	-	8	19.04		
	Dry mix	-	-	6	2.25	-	-	6	2.68		
*B. sulcata*	Natural milk	23	32.86	-	-	18	36.73	-	-	0.74	0.000
	Green fodder	-	-	13	20.63	-	-	9	21.42		
	Dry mix	-	-	6	9.52	-	-	3	7.14		
*Cryptosporidium* species	Breast milk	7	10	-	-	5	10.20	-	-	0.55	0.000
	Green fodder	-	-	2	3.17	-	-	2	4.76		
	Dry mix	-	-	1	1.58	-	-	1	2.38		
*T. vitulorum*	Breast milk	14	20	-	-	6	12.24	-	-	0.55	0.000
	Green fodder	-	-	9	14.28	-	-	7	16.67		
	Dry mix	-	-	6	9.52	-	-	1	2.38		
*Moneizia* species	Breast milk	-	-	-	-	-	-	-	-	0.32	No value
	Green odder	-	-	5	7.93	-	-	4	9.52		
	Dry mix	-	-	3	4.76	-	-	1	2.38		

Data are presented as number of positive animals, with prevalence rate in parentheses; * *p*-value ≤ 0.05 is significant.

**Table 6:** *B. sulcata* cyst count before and after treatment in the drug-treated, garlizine-treated, and untreated control groups (for two weeks). CPG: cysts per gram of feces.

Treatment Type	CPG before Treatment	SEM	CPG after Treatment	SEM	Efficacy %
Group A	870 ^a^	98.234	32 ^a^	14.543	96.44
Group B	818 ^a^	77.097	11 ^a^	14.543	98.77
Group C	826 ^a^	61.204	250 ^b^	57.008	72.22
Group D	930 ^a^	61.652	900 ^c^	121.652	0.00
*p*-value	0.856		0.000 *		

Values are presented as means (n = 5) and standard error of the mean (SEM); *, a, b, and, c are means within the same column (in each trial, independently), and superscripts represent significant differences (*p* < 0.05).

## Supplementary Materials

The following are available online at https://www.mdpi.com/2076-2615/9/5/259/s1, Table S1. Species-wise prevalence rate of Eimeria spp. in cattle and buffalo calves, Table S2. Prevalence of internal parasites in diarrheic calves according to the types of infections, Table S3. Prevalence rate of parasitic diarrhea in relation to gender, Table S4. Prevalence rate of parasitic diarrhea in relation to housing system, Table S5. Seasonal prevalence rate of internal parasites among diarrheic calves, Figure.S1. Buxtonella sulcate. (A) Trophozoite form (low magnification). (B) Trophozoite (Higher magnification). (C) Cyst form, Figure S2. Calves suffering from diarrhea, (A) Arched back position due to B. sulcate, (B) Bloody diarrhea due to Eimeria spp, (C) Toxocara vitulorum protruded from anal opening in calf.
