# Supplementary material for: Diversity of Parasitic Diarrhea Associated with Buxtonella Sulcata in Cattle and Buffalo Calves with Control of Buxtonellosis
Source: Animals (Basel). 2019 May 21;9(5):259. doi: 10.3390/ani9050259 (PMC6562938; doi:10.3390/ani9050259)
Supplement: Supplementary file 1 [file animals-09-00259-s001.pdf]

Supplementary data

Table S1. Species-wise prevalence rate of *Eimeria* spp. in cattle and buffalo calves

Age groups	Species	Prevalence of *Eimeria* spp.					*P*- value
		E. bovis	*E. zurenii*	*E. ellipsoidalis*	*E. alabamensis*	*E. bukidnonensis*	
Suckling calves (1d-60d)	Cattle (70)	26 (37.14%)	26 (37.14%)	23(32.86%)	21 (30%)	16 (22.86%)	0.595
	Buffalo (49)	20 (40.82%)	16 (32.65%)	12 (24.49%)	12 (24.49%)	7 (14.29%)	
Post weaning calves (below 6m)	Cattle (63)	18 (28.57%)	18 (28.57%)	16 (25.40%)	14 (22.22%)	11 (17.46%)	0.810
	Buffalo (42)	14 (33.33%)	11 (26.19%)	8 (19.04%)	8 (19.04%)	5 (11.90%)	
Total	224	78 (34.82%)	71 (31.69%)	59 (26.34%)	55(24.55 %)	39 (17.41%)	

*P-value > 0.05 is non-significant (NS)

Table S2. Prevalence of internal parasites in diarrheic calves according to the types of infections

Type of Parasites	No. of Mono-infections	No. of mixed infections				
		Eimeria spp.	*B. sulcata*	*Cryptosporidium* spp.	*T. vitulorum*	*Moneizia* spp.
Eimeria spp.	37 (16.51%)	-	21 (9.38%)	5 (2.23%)	9 (4.02)	6 (2.68%)
B.sulcata	35 (15.62%)		21 (9.38%)	3 (1.34%)	12(1.09%)	1 (0.45%)
Cryptosporidium spp.	10 (4.46%)	5 (2.23%)	3 (1.34%)	-	-	-

T. vitulorum	17(7.59%)	9 (4.02)	12(5.36%)	-		5 (2.23%)
Moneizia spp.	1(0.45%)	6 (2.68%)	1 (0.45%)	-	5(2.23%)	-

Table S3. Prevalence rate of parasitic diarrhea in relation to gender

Internal Parasites	Sex	Cow calves				Buffalo calves				*P*-value*
		Suckling calves (70)		Post weaning calves (63)		Suckling calves(49)		Post weaning calves(42)		
		No.	%	No.	%	No.	%	No.	%	
Eimeria spp.	Male	16	22.85	11	17.46	12	24.49	9	21.43	0.822
	Female	10	14.29	7	11.11	8	16.33	5	11.90	
Buxtonella sulcata	Male	15	21.43	13	20.63	11	22.45	7	16.67	0.913
	Female	8	11.43	6	9.52	7	14.29	5	11.90	
Cryptosporidium spp.	Male	3	4.29	1	1.59	2	4.08	1	2.38	
	Female	4	5.71	2	3.17	3	6.12	2	4.76	0.706
Toxocara vitulorum	Male	5	7.14	7	11.11	2	4.08	3	7.14	0.459
	Female	9	12.86	8	12.70	4	8.16	5	11.90	
Moneizia spp.	Male	-	-	5	7.94	-	-	3	7.14	
	Female	-	-	3	4.76	-	-	2	4.76	No value

*P-value > 0.05 is non-significant (NS)

Table S4. Prevalence rate of parasitic diarrhea in relation to housing system

Internal Parasites	Housing system	Cow calves				Buffalo calves				P-value*
		Suckling calves(70)		Post weaning calves (63)		Suckling calves(49)		Post weaning calves(42)		
		No.	%	No.	%	No.	%	No.	%	
Eimeria spp.	Paved	10	14.29	7	11.11	8	16.33	5	11.90	0.432
	Dirt yard	16	22.86	11	17.46	12	24.49	9	21.43	
Buxtonella sulcata	Paved	3	4.29	6	9.52	7	14.29	4	9.52	0. 749
	Dirt yard	15	21.43	13	20.63	11	22.45	8	19.05	
Cryptosporidium spp.	Paved	2	2.86	1	1.59	2	4.08	1	2.38	0.923
	Dirt yard	5	7.14	2	3.17	3	6.12	2	4.76	
Toxocara vitulorum	Paved	6	8.57	6	9.52	2	4.08	3	7.14	0.924
	Dirt yard	8	11.43	9	14.29	4	8.16	5	11.90	
	Paved	-	-	4	6.35	-	-	2	4.76	No value

Moneizia spp.	Dirt yard	-	-	4	6.35	-	-	3	7.14

*P-value > 0.05 is non-significant (NS)

Table S5. Seasonal prevalence rate of internal parasites among diarrheic calves

Internal Parasites	Season	Cow calves				Buffalo calves				Total %	*P*-value*
		Suckling calves(70)		Post weaning calves (63)		Suckling calves(49)		Post weaning calves(42)			
		No.	%	No.	%	No.	%	No.	%		
Eimeria spp.	Winter	4	5.71	4	6.35	3	6.12	2	4.76	13 (5.80%)	0.924
	Spring	9	12.86	5	7.94	7	14.29	6	14.29	27 (12.05%)	
	Summer	5	7.14	4	6.35	4	8.61	3	7.14	16 (7.14%)	
	Autumn	8	11.43	5	7.94	6	12.24	3	7.14	22 (9.82%)	
Buxtonella sulcata	Winter	5	7.14	5	7.94	3	6.12	3	7.14	16 (7.14%)	0.841
	Spring	8	11.43	6	9.52	7	14.29	4	9.52	25 (11.16%)	

	Summer	4	5.71	3	4.76	3	6.12	2	4.76	12 (5.36%)	
	Autumn	6	8.57	5	7.94	5	10.20	3	7.14	19 (8.48%)	
Cryptosporidium spp.	Winter	2	2.86	0	0	1	2.04	1	2.38	4 (1.79%)	0.437
	Spring	2	2.86	2	3.17	1	2.04	1	2.38	6 (2.68%)	
	Summer	1	1.43	0	0	1	2.04	0	0	2 (0.89%)	
	Autumn	2	2.86	1	1.59	2	4.08	1	2.38	6 (2.68%)	
Toxocara vitulorum	Winter	5	7.14	5	7.94	3	6.12	2	4.76	15 (6.70%)	0.922
	Spring	3	4.29	4	6.35	1	2.04	1	2.38	9 (4.02%)	
	Summer	2	2.86	1	1.59	1	2.04	1	2.38	5 (2.23%)	
	Autumn	3	4.29	2	3.17	1	2.04	1	2.38	8 (3.57%)	
Moneizia spp.	Winter	0	0	3	4.76	0	0	3	7.14	6 (2.68%)	No value
	Spring	0	0	3	4.76	0	0	3	7.14	6(2.68%)	
	Summer	0	0	2	3.17	0	0	1	2.38	3 (1.34%)	
	Autumn	0	0	2	3.17	0	0	2	4.76	4 (1.79%)	

*P-value > 0.05 is non-significant (NS)

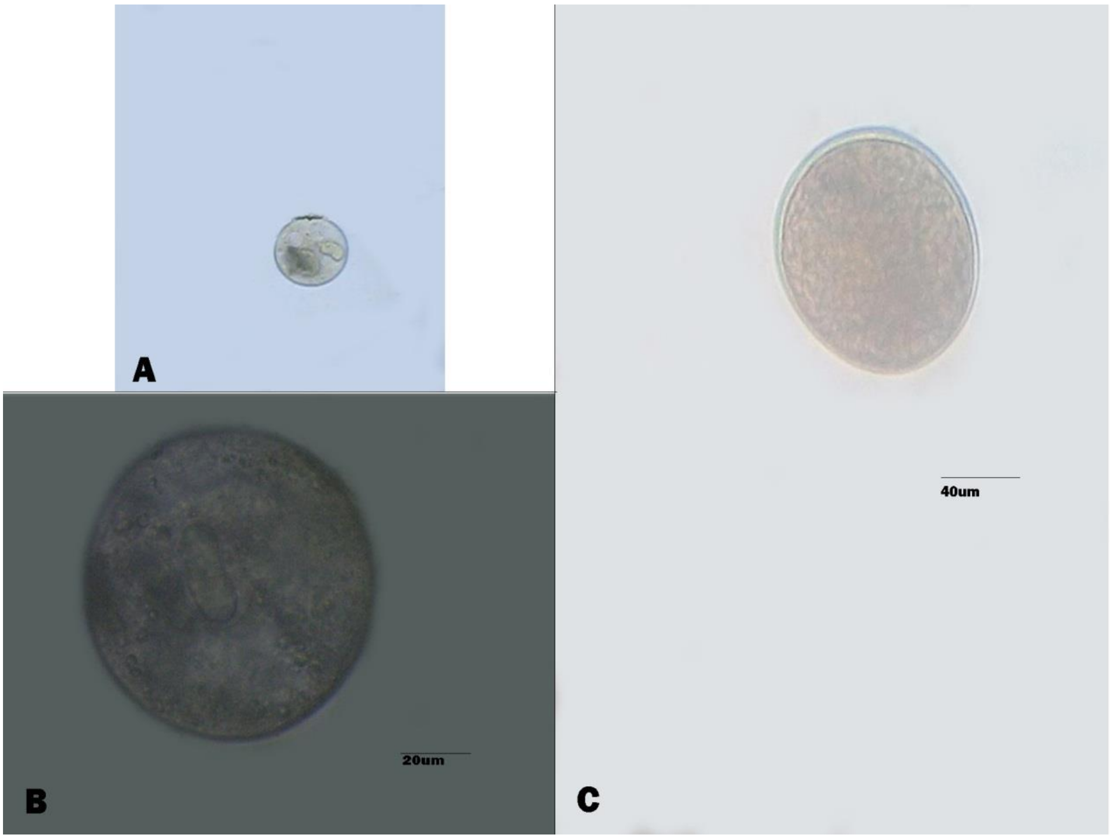

Figure.S1. *Buxtonella sulcate*. A. Trophozoite form (low magnification). B. Trophozoite (Higher magnification). C. Cyst form

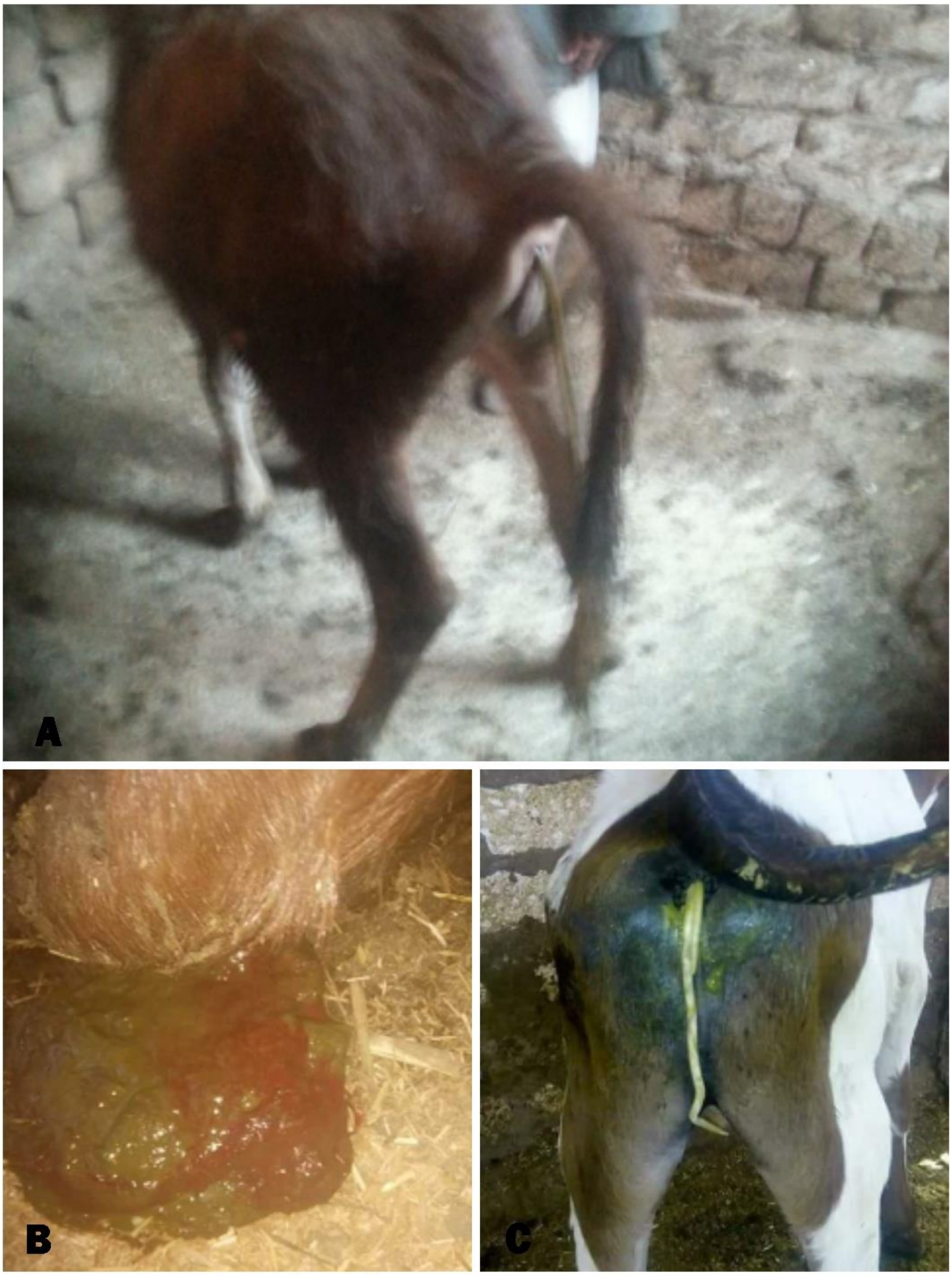

Figure. S2. Calves suffering from diarrhea, (A) Arched back position due to *B. sulcate*, (B) Bloody diarrhea due to *Eimeria* spp, (C) *Toxocara vitulorum* protruded from anal opening in calf
